# External validation of pathological T adjustments in the ninth edition of the pleural mesothelioma tumor-node-metastasis classification

**DOI:** 10.3389/fonc.2025.1557097

**Published:** 2025-09-10

**Authors:** Zheng Liu, Jingsheng Cai, Yun Li, Wenhan Weng

**Affiliations:** ^1^ Thoracic Oncology Institute and Research Unit of Intelligence Diagnosis and Treatment in Early Non-small Cell Lung Cancer, Peking University People’s Hospital, Beijing, China; ^2^ Department of Thoracic Surgery, Peking University People’s Hospital, Beijing, China; ^3^ Institute of Advanced Clinical Medicine, Peking University, Beijing, China; ^4^ Department of Thoracic Surgery of Tangdu Hospital, Fourth Military Medical University, Xi'an, China; ^5^ Department of Thoracic Surgery, Sun Yat-sen University Cancer Center, Guangzhou, China

**Keywords:** pleural mesothelioma, TNM classification, T category, fissural invasion, IASLC

## Abstract

**Introduction:**

In the ninth edition pleural mesothelioma (PM) pathological (p) T staging, patients with fissural invasion are upgraded from T1 to T2. This study aimed to externally validate this staging modification.

**Methods:**

Resected pT1/2 PM patients were selected. The Kaplan–Meier method was applied to assess survival differences, and propensity score matching was utilized to balance baseline characteristics. Univariable and multivariable Cox analyses were conducted to determine prognostic factors. Multiple model parameters were used to evaluate the performance of the ninth and eighth edition pT staging in distinguishing between T1 and T2 patients.

**Results:**

A total of 818 eligible patients were included, among whom 325 initially classified as T1 were reclassified as T2 due to fissural invasion, resulting in 57 patients remaining with pT1 disease. Survival analyses demonstrated that both before and after matching, the ninth edition T staging effectively differentiated between T1 and T2 patients, whereas the eighth edition did not perform as satisfactorily in distinguishing between the groups. Cox regression analyses further confirmed that the ninth edition T staging was a strong prognostic factor, whereas the eighth edition T staging was not prognostic. Lastly, model parameter results indicated that the ninth edition T staging performed better in distinguishing between T1 and T2 patients compared to the eighth edition.

**Discussion:**

Our study provided validation and endorsement for the revisions implemented in the ninth edition pT staging, specifically the reclassification of patients with fissural invasion from pT1 to pT2. This research contributed to the precise staging of PM patients.

## Graphical Abstract

Our research confirmed and supported the changes made in the ninth edition pT staging, particularly the reclassification of patients with fissural invasion from pT1 to pT2. TNM, tumor-node-metastasis.

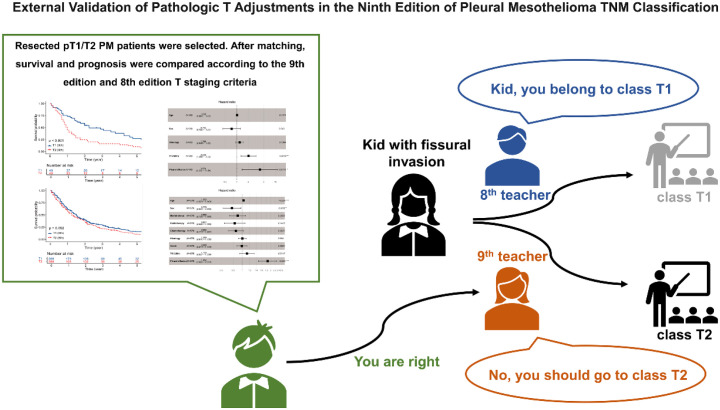

## Introduction

Pleural mesothelioma (PM) is a malignant tumor derived from mesothelial cells lining the pleura, with a relatively low incidence predominantly associated with asbestos exposure (1–3). Due to its insidious onset, most patients present with overt clinical symptoms, often at an advanced stage, with a median 5-year survival rate of approximately 10% (4, 5). In 2024, the International Association for the Study of Lung Cancer (IASLC) Mesothelioma Staging Project introduced the revised T descriptors in the ninth edition of the PM tumor-node-metastasis (TNM) classification (6). The updated staging incorporates maximum pleural thickness in the clinical T descriptors, while the pathological (p) ones show minimal changes from those in the eighth edition (7), except for elevating fissural invasion from pT1 to pT2 (6). In the pT descriptor of the ninth edition, T1 is defined as a tumor confined to the ipsilateral pleura without fissural involvement, while T2 is defined as a tumor involving the ipsilateral pleura with any of the following: involvement of the fissure, invasion of the ipsilateral lung parenchyma, or non-transmural invasion of the diaphragm (6).

Considering the prognostic value of the pTNM staging of PM, it is essential to validate the revision introduced in the newly released edition, as external validation with independent cohorts is currently lacking. Herein, this study included patients with pT1–2 PM from the Surveillance, Epidemiology, and End Results (SEER) database to assess the prognostic performance of the ninth edition versus the eighth edition T1/2 descriptors. We aimed to externally validate the modification in pT staging and further substantiate the rationale for the change.

## Methods

### Study subject

**Figure 1:**
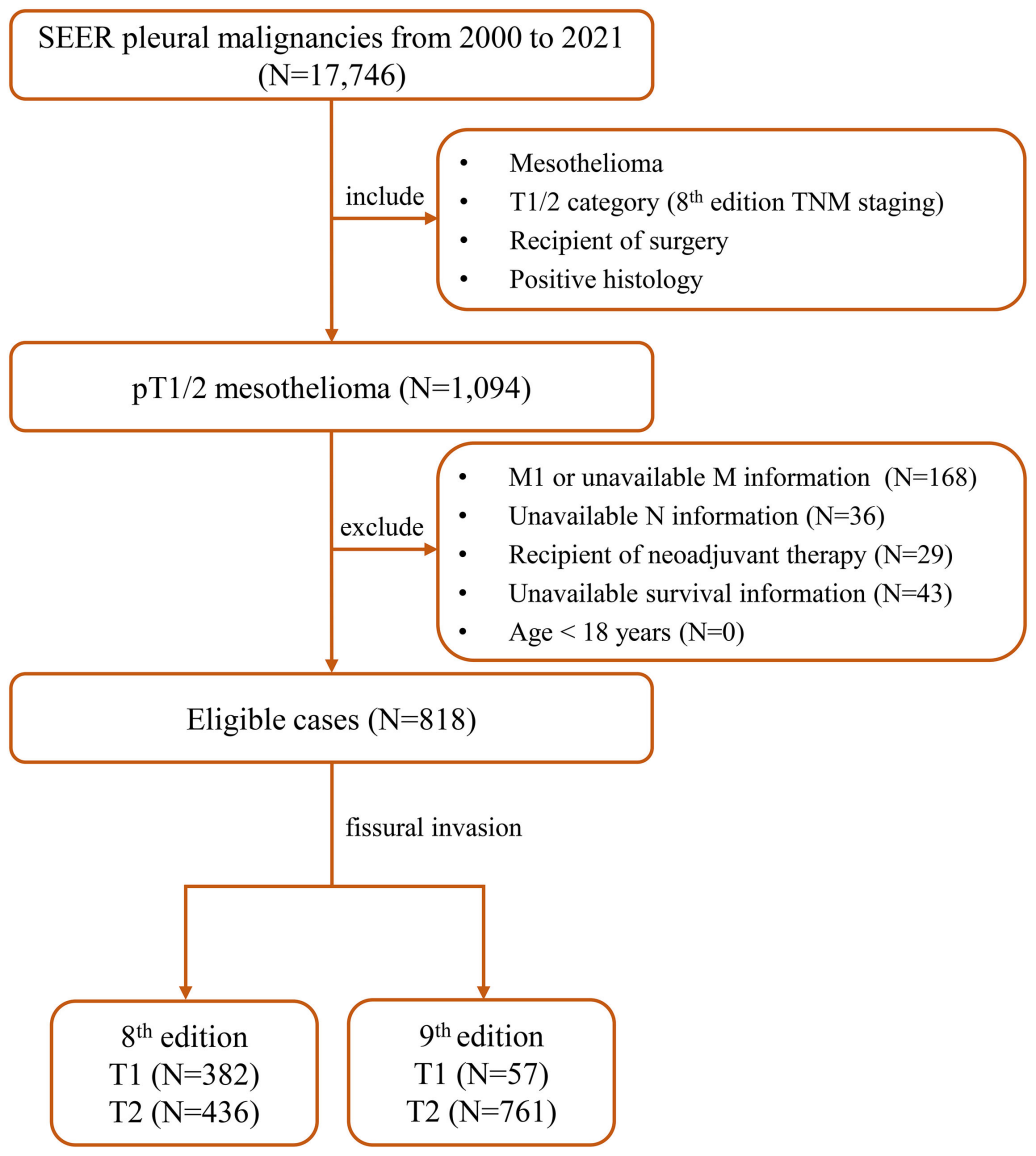
The detailed patient selection process. SEER, the Surveillance, Epidemiology, and End Results; TNM, tumor-node-metastasis.

Pleural malignancies diagnosed between 2000 and 2021 were retrospectively analyzed using data from the SEER program (https://seer.cancer.gov). In this study, inclusion criteria consisted of the following: 1) diagnosis of PM (site codes C34.0–C34.9 and C38.4 and ICD-O-3 histology/behavior codes 9050–9055), 2) T1/2 [as per the eighth edition TNM staging (7)), 3] underwent surgery, and 4) positive histology. Exclusion criteria included the following: 1) M1 diseases or unavailable M information, 2) unavailable N information, 3) receipt of neoadjuvant therapy, 4) unavailable survival information, and 5) age under 18 years. The eligible T1/2 cases were reclassified using the ninth edition pT staging criteria (6). Positive fissural invasion was defined according to CS Extension code 140. The detailed patient selection process is illustrated in **Figure 1**.

### Ethic statement

Due to the retrospective nature and the use of anonymized data, ethical approval from an institutional review board was deemed unnecessary, and individual patient informed consent was also waived. This study adhered to the principles outlined in the Helsinki Declaration.

### Data collection

Data pertaining to the included patients were retrieved from the electronic database maintained by the SEER program, capturing various epidemiological characteristics such as age, sex, race, marital status, median income, residence, year of diagnosis, survival time, status, and cause of death. Additionally, tumor features including laterality, histology, grade, presence of pleural effusion (CS Site-Specific Factor 1 codes 010, 020, and 030), T category, N category, and treatment details (such as surgery, radiotherapy, and chemotherapy) were also collected. The primary endpoints of this study were overall survival (OS) and cancer-specific survival (CSS). OS was defined as the interval from the date of diagnosis to the date of death or last follow-up. CSS was defined as the interval from the date of diagnosis to the date of death due to PM or the last follow-up. Missing values for baseline covariates were addressed using dummy variables, and patients with incomplete survival data were excluded from the analysis.

### Statistical analysis

Data extraction from the SEER program was conducted using the SEER*Stat software version 8.4.3 (https://seer.cancer.gov/seerstat). Statistical analyses were carried out using the IBM SPSS Statistics software version 25.0 (IBM Corp., Armonk, NY, USA) and the R software version 4.1.1 (The R Foundation for Statistical Computing, Vienna, Austria; https://www.r-project.org). Categorical variables were presented as counts and percentages, while continuous variables were summarized using median and interquartile range (IQR). Survival curves were generated using the Kaplan–Meier method (8), and differences in survival among groups were assessed using the log-rank test. To address potential bias due to unbalanced baseline characteristics, one-to-one propensity score matching (PSM) (9) was performed using the R package “MatchIt” (method=nearest, replace=FALSE), and covariate balance was assessed using standardized mean difference (SMD). A caliper distance of 0.01 was used for matching pairs in both the ninth edition T1 and T2 and the eighth edition T1 and T2 groups. Univariable and multivariable Cox proportional hazards analyses (forced entry method) were performed to identify final prognostic factors (variables with p-value < 0.1 in the univariable analyses were entered into multivariable analyses). The results of multivariable Cox analyses were visually presented using forest plots. The discriminatory performance of the ninth edition T1/2 staging compared to the eighth edition in predicting patient prognosis was assessed using the concordance index (C-index), Akaike information criterion (AIC), Bayesian information criterion (BIC), and net reclassification improvement (NRI) index. Statistical significance was defined as two-sided p-values < 0.05.

## Results

### Baseline characteristics

Between 2000 and 2021, a comprehensive review was conducted on 17,746 cases of pleural malignancies. After applying predefined inclusion and exclusion criteria, 818 cases of T1/2 PM were identified for detailed analysis. The baseline characteristics of the entire cohort are detailed in **Table 1**. The median age of the cohort was 71 years (IQR, 64–78 years), with a predominant male distribution (616/818, 75.3%) and a majority of Caucasian ethnicity (757/818, 92.5%). Approximately half of the patients underwent chemotherapy (441/818, 53.9%), while radiotherapy (161/818, 19.7%) was less commonly administered. In terms of the ninth edition T staging, 57 cases were categorized as T1 and 761 as T2, compared to 382 cases classified as T1 and 436 cases as T2 according to the eighth edition criteria. Pleural effusion was a common finding, observed in the majority of patients (566/818, 69.2%).

**Table 1:** The clinical–pathological characteristics of the included pleural mesothelioma patients.

Characteristic	Entire cohort (N = 818)
Age, years	
Continue (median, IQR)	71.00 (64.00–78.00)
Sex	
Male	616 (75.3)
Female	202 (24.7)
Race	
Caucasian	757 (92.5)
African	32 (3.9)
Other	29 (3.5)
Marital status	
Other	266 (32.5)
Married	552 (67.5)
Income, dollars	
≤80,000	499 (61.0)
>80,000	319 (39.0)
Residence	
Urban	739 (90.3)
Rural	79 (9.7)
Year of diagnosis	
2000–2010	367 (44.9)
2011–2021	451 (55.1)
Radiotherapy	
Not performed	657 (80.3)
Performed	161 (19.7)
Chemotherapy	
Not performed	377 (46.1)
Performed	441 (53.9)
Histology	
Sarcomatoid	78 (9.5)
Epithelioid	405 (49.5)
Biphasic	103 (12.6)
Mesothelioma NOS	232 (28.4)
Grade	
Well	24 (2.9)
Moderate	11 (1.3)
Poor	39 (4.8)
Undifferentiated	14 (1.7)
Unknown	730 (89.2)
Laterality	
Right	484 (59.2)
Left	323 (39.5)
Other	11 (1.3)
T category (9th)	
1	57 (7.0)
2	761 (93.0)
T category (8th)	
1	382 (46.7)
2	436 (53.3)
N category	
0	643 (78.6)
1	161 (19.7)
2	14 (1.7)
Pleural effusion	
Without	252 (30.8)
With	566 (69.2)

IQR, interquartile range; NOS, not otherwise specified.

To balance the baseline characteristics, T1 and T2 PM patients underwent PSM. Post-matching, there were 49 pairs in the ninth edition T1 and T2 matched group and 289 pairs in the eighth edition T1 and T2 matched group (**Supplementary Table S1**).

### Survival analysis

**Figure 2:**
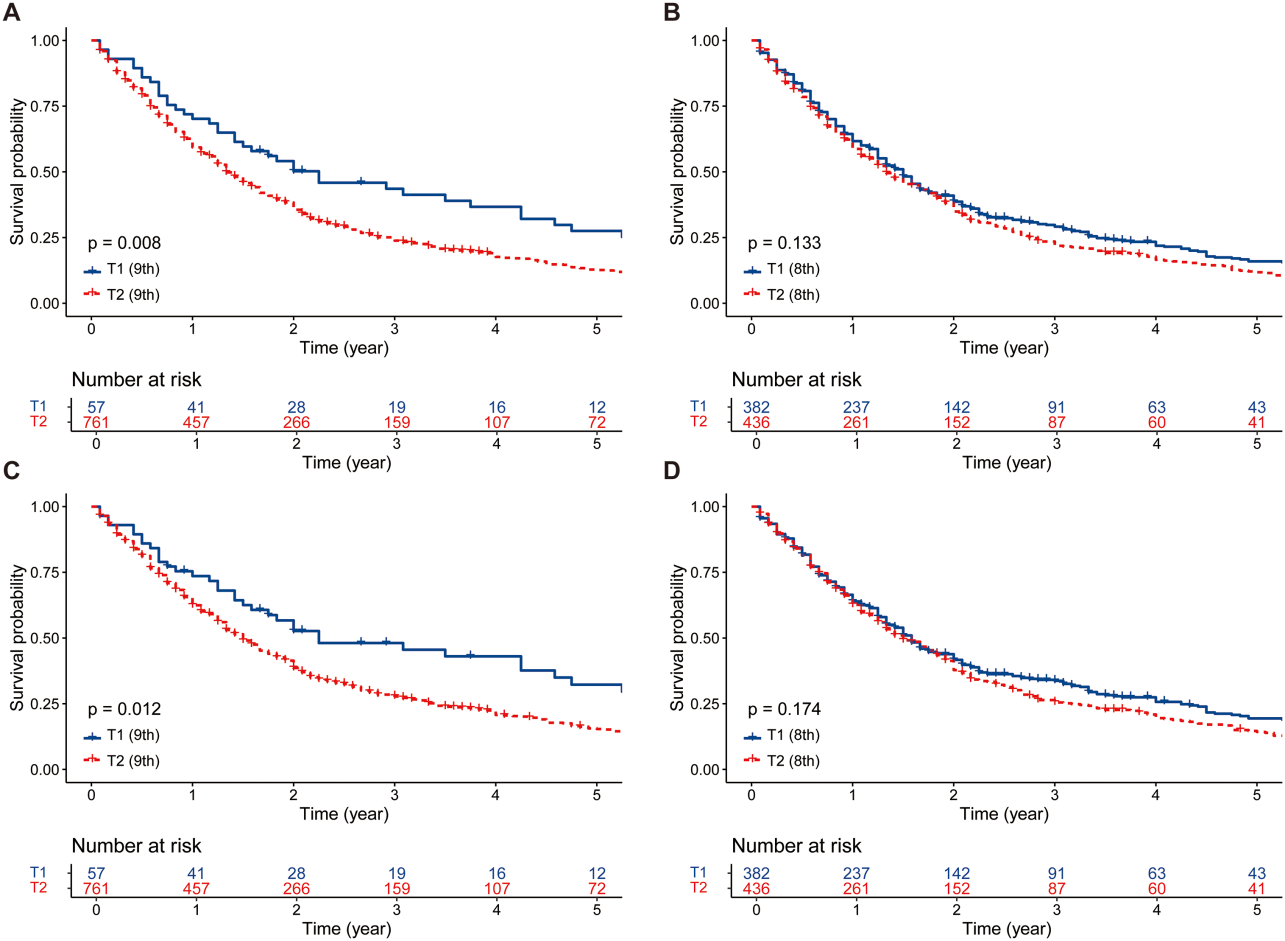
The survival comparisons between T1 and T2 patients before PSM. **(A)** OS: T1 (ninth edition) vs. T2 (ninth edition). **(B)** OS: T1 (eighth edition) vs. T2 (eighth edition). **(C)** CSS: T1 (ninth edition) vs. T2 (ninth edition). **(D)** CSS: T1 (eighth edition) vs. T2 (eighth edition). PSM, propensity score matching; OS, overall survival; CSS, cancer-specific survival.

Regarding OS, in the ninth edition T cohort, patients classified as T1 exhibited significantly superior OS compared to those classified as T2 (5-year OS rate, 27.5% vs. 12.7%, p = 0.008, **Figure 2A**). Conversely, in the eighth edition T cohort, there was no significant difference in OS between T1 and T2 patients (5-year OS rate, 15.9% vs. 11.8%, p = 0.133, **Figure 2B**). In the analysis of CSS, similar outcomes to OS were also observed (ninth edition T, 5-year CSS rate, 32.3% vs. 15.4%, p = 0.012, **Figure 2C**; eighth edition T, 5-year CSS rate, 19.4% vs. 14.3%, p = 0.174, **Figure 2D**).

**Figure 3:**
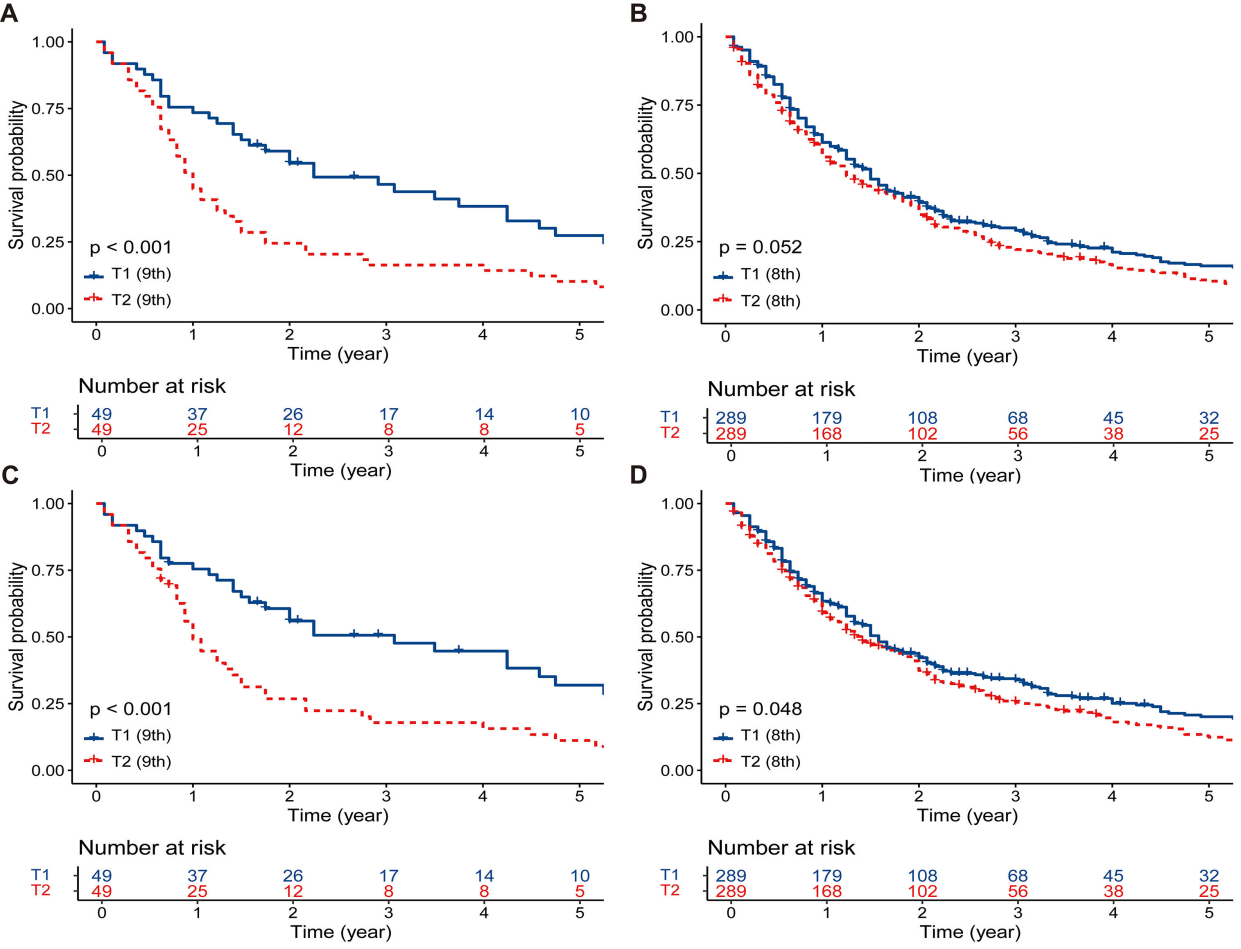
The survival comparisons between T1 and T2 patients after PSM. **(A)** OS: T1 (ninth edition) vs. T2 (ninth edition). **(B)** OS: T1 (eighth edition) vs. T2 (eighth edition). **(C)** CSS: T1 (ninth edition) vs. T2 (ninth edition). **(D)** CSS: T1 (eighth edition) vs. T2 (eighth edition). PSM, propensity score matching; OS, overall survival; CSS, cancer-specific survival.

Following PSM, the survival advantage of T1 patients over T2 patients persisted in the ninth edition T group (5-year OS rate, 27.4% vs. 10.2%, p < 0.001, **Figure 3A**; 5-year CSS rate, 31.9% vs. 11.2%, p < 0.001, **Figure 3C**). In the eighth edition T group, the survival of T1 patients was marginally better than that of T2 patients (5-year OS rate, 16.1% vs. 10.5%, p = 0.052, **Figure 3B**; 5-year CSS rate, 20.1% vs. 12.4%, p = 0.048, **Figure 3D**).

### Prognosis analysis

**Figure 4:**
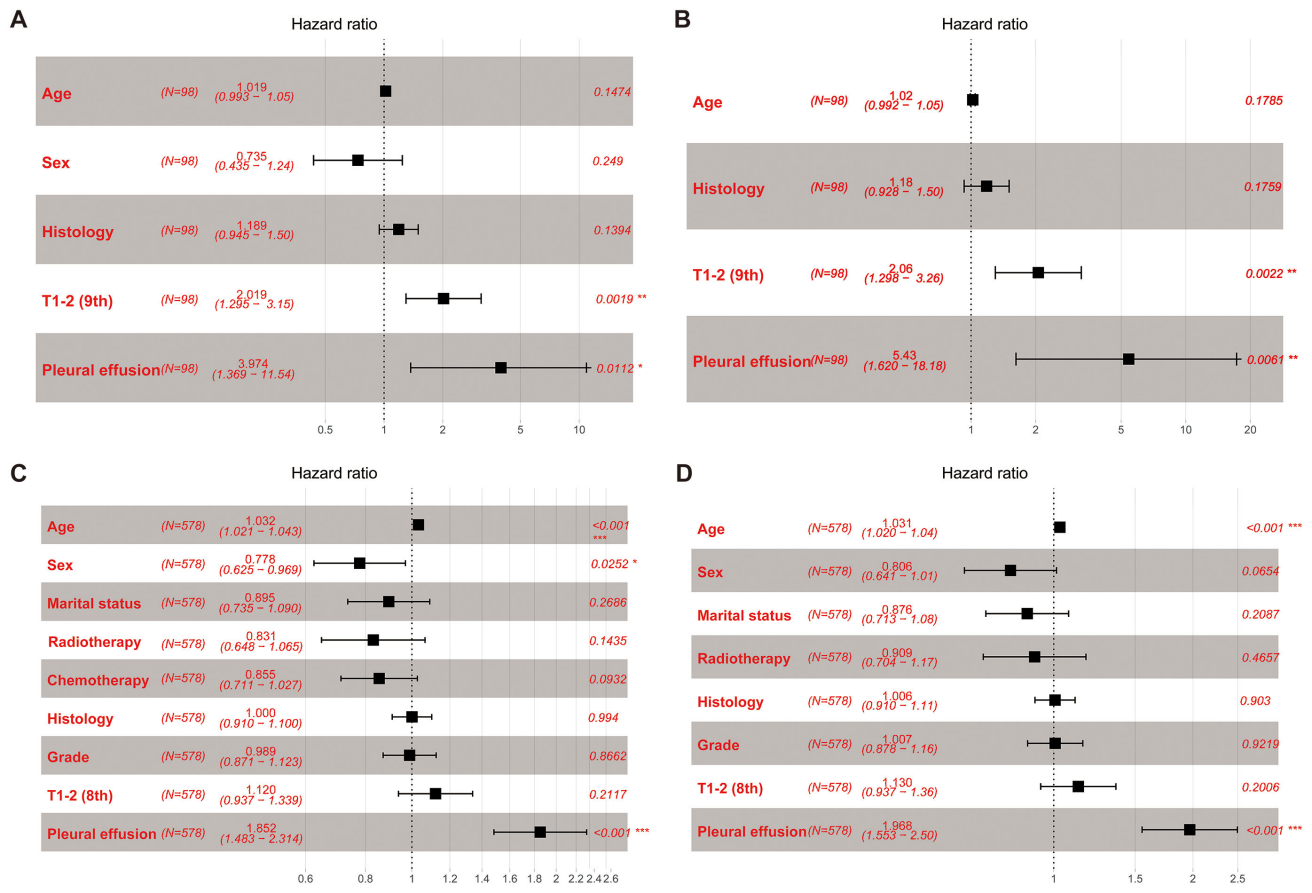
Forest plots. **(A)** OS: multivariable Cox analyses within the T category (ninth edition) cohort. **(B)** CSS: multivariable Cox analyses within the T category (ninth edition) cohort. **(C)** OS: multivariable Cox analyses within the T category (eighth edition) cohort. **(D)** CSS: multivariable Cox analyses within the T category (eighth edition) cohort. OS, overall survival; CSS, cancer-specific survival.

In the ninth edition T matching cohort, univariable Cox analyses identified T category (ninth) as a potential prognostic factor for OS (T1 vs. T2, hazard ratio (HR) = 1 vs. 2.092 [1.350–3.243], p = 0.001, **Supplementary Table S2**) and CSS (T1 vs. T2, HR = 1 vs. 2.205 [1.393–3.491], p = 0.001, **Supplementary Table S2**). Subsequent multivariable Cox analyses incorporating T category (ninth) confirmed its status as an independent prognostic factor (OS, HR = 1 vs. 2.019 [1.295–3.150], p = 0.002, **Figure 4A**; CSS, HR = 1 vs. 2.060 [1.298–3.260], p = 0.002, **Figure 4B**).

In the eighth edition T matching cohort, univariable Cox analyses indicated that T category (eighth) may be a potential prognostic factor for OS (T1 vs. T2, HR = 1 vs. 1.186 [0.995–1.415], p = 0.057, **Supplementary Table S3**) and CSS (T1 vs. T2, HR = 1 vs. 1.200 [0.997–1.443], p = 0.053, **Supplementary Table S3**). Upon inclusion of T category (eighth) in the multivariable Cox analyses, data showed that T category (eighth) was not an independent prognostic factor for survival (OS, T1 vs. T2, HR = 1 vs. 1.120 [0.937–1.339], p = 0.212, **Figure 4C**; CSS, T1 vs. T2, HR = 1 vs. 1.130 [0.937–1.360], p = 0.201, **Figure 4D**).

### Predictive performance: ninth T1/2 vs. eighth T1/2

The performance of the ninth edition T1/2 and eighth edition T1/2 was assessed using several indices, including C-index, AIC, BIC, and NRI (**Table 2**). When considering OS, the C-index of the ninth edition T1/2 (0.514 [0.502–0.526]) slightly outperformed that of the eighth edition T1/2 (0.513 [0.492–0.534]), while the AIC was lower for the ninth edition T1/2 (8,180.463) compared to the eighth edition T1/2 (8,186.009). Similarly, the BIC was smaller for the ninth edition T1/2 (8,185.170) than for the eighth edition T1/2 (8,190.716). Using a risk cutoff of 0.8–0.85, the 5-year NRI+ was 0.396 for the ninth edition versus the eighth edition.

**Table 2:** Comparative performance of the T1/2 categories in the ninth and eighth editions for predicting patient prognosis.

Index	OS		CSS	
	9th edition	8th edition	9th edition	8th edition
C-index	0.514 (0.502–0.526)	0.513 (0.492–0.534)	0.514 (0.502–0.526)	0.509 (0.487–0.531)
AIC	8,180.463	8,186.009	7,373.063	7,378.198
BIC	8,185.170	8,190.716	7,377.770	7,382.905
5-year NRI	9th vs. 8th: NRI+ 0.396		9th vs. 8th: NRI+ 0.339	

OS, overall survival; CSS, cancer-specific survival; C-index, concordance index; AIC, Akaike information criterion; BIC, Bayesian information criterion; NRI, net reclassification improvement.

Regarding CSS, the C-index of the ninth edition T1/2 [0.514 (0.502–0.526)] was superior to that of the eighth edition T1/2 [0.509 (0.487–0.531)], and the AIC was lower for the ninth edition T1/2 (7,373.063) compared to the eighth edition T1/2 (7,378.198). Similarly, the BIC was smaller for the ninth edition T1/2 (7,377.770) than for the eighth edition T1/2 (7,382.905). Using a risk cutoff of 0.8–0.85, the 5-year NRI+ was 0.339 for the ninth edition versus the eighth edition.

## Discussion

In this study, we included patients diagnosed with pT1/2 PM and evaluated the prognostic performance of the ninth edition pT1/2 relative to the eighth edition pT1/2, considering both OS and CSS. Our data demonstrated that compared to the eighth edition T staging, the ninth edition T staging showed improved ability to differentiate prognosis between T1 and T2 patients. These findings further supported the rationale behind the adjustments introduced in the current ninth edition pT staging.

Previous research has shown that fissural invasion is closely related to patient prognosis (10). In their study, the median survival time for patients with fissural invasion was 17 months, significantly lower than the 25.8 months for patients without fissural invasion (10). This suggested that fissural invasion may be a critical factor for upstaging. The ninth edition PM T staging also recognized the prognostic significance of fissural invasion. Therefore, all pT descriptors were retained, but fissural invasion was reclassified as part of the pT2 category (6). However, the database of the IASLC project included only 60 pT1 patients and 129 pT2 patients, of which only nine were upstaged from T1 to T2 due to fissural invasion (6, 11). Given the critical importance of accurate TNM staging in predicting patient prognosis, it is essential to validate the change using external databases.

Our study included PM patients from the U.S. SEER database spanning 2000 to 2021, offering a large and diverse cohort that is representative of the population. Following rigorous inclusion and exclusion criteria, we identified 818 patients with pT1–2 disease, of which 325 patients initially classified as pT1 were reclassified as pT2 due to fissural invasion, leaving 57 patients with pT1 disease. This reflected the typical presentation of PM, characterized by a subtle onset where many patients present at an advanced stage beyond localized disease (4, 5). Our findings demonstrated that the ninth edition pT staging effectively differentiated between pT1 and pT2 patients both before and after baseline matching, whereas the eighth edition T staging exhibited inferior performance in patient stratification. Multivariable Cox regression analysis provided further support for these results. Additionally, multiple model indices confirmed the superior predictive accuracy of the ninth edition pT staging compared to the eighth edition. Our perspective underscored the importance of differentiating PM with or without fissural invasion. Fissural invasion may signify tumor invasiveness, which is closely associated with poor patient prognosis.

In the context of various cancers, focal tumor infiltration has evolved as a critical determinant for tumor staging advancement. For example, in non-small cell lung cancer (NSCLC), tumors measuring less than 3 cm that exhibit visceral pleural invasion are categorized as T2a (12); the presence of lymphovascular invasion is regarded as a high-risk prognostic factor associated with unfavorable outcomes in NSCLC (13, 14). This underscores the importance of considering specific tumor characteristics in staging and predicting the course of disease. The ninth edition PM T staging introduces fissural invasion as a determinant for T-stage upstaging. This holds significant importance in accurately defining patient TNM staging and more precisely predicting patient prognosis.

Our study has several limitations worth noting. First, due to the absence of data on maximum pleural thickness in the SEER database, we were unable to validate the accuracy of cT staging. Given that the majority of PM patients did not undergo surgical treatment, this underscores the importance of clinical staging. Therefore, further comprehensive data will be needed to validate clinical staging. Second, despite strict patient selection criteria, the number of pT1 patients remains limited at only 57 cases, which could potentially weaken statistical power. However, given the low incidence of PM, conducting large-scale analyses on pT1 patients presents challenges, and future multicenter data may be required to further substantiate our findings. Third, specific clinical details relevant to PM, such as asbestos exposure, were unavailable in the SEER database, thereby limiting our understanding of their impact on patient prognosis. Fourth, information regarding the type of surgical procedure (e.g., extrapleural pneumonectomy or pleurectomy/decortication) is not provided in the SEER dataset. As such, we were unable to evaluate whether surgical type influenced survival outcomes. We recognize this as a meaningful limitation, as the surgical approach may reflect both patient fitness and tumor burden. Finally, the retrospective nature of our study introduces inherent risks of selection bias and unmeasured confounders. For instance, patients who underwent surgical resection may represent a fitter subgroup with better performance status or disease control after induction therapy, which could have positively influenced survival outcomes. However, due to the lack of information on preoperative clinical status and induction treatment in the SEER database, we were unable to assess or adjust for this potential confounding effect.

In summary, our study validated and supported the modifications introduced in the ninth edition PM pT staging, particularly the reclassification of patients with fissural invasion from pT1 to pT2. Our research contributed to the accurate staging of PM patients.

## Data Availability

The original contributions presented in the study are included in the article/**Supplementary Material**. Further inquiries can be directed to the corresponding author.
