# Supplementary material for: External validation of pathological T adjustments in the ninth edition of the pleural mesothelioma tumor-node-metastasis classification
Source: Front Oncol. 2025 Sep 10;15:1557097. doi: 10.3389/fonc.2025.1557097 (PMC12457411; doi:10.3389/fonc.2025.1557097)
Supplement: Supplementary file 1 [file Table1.docx]

**Table S1. Baseline characteristics of T1 and T2 patients after PSM**

| Characteristics | 9^th^ edition | | | 8^th^ edition | | |
| --- | --- | --- | --- | --- | --- | --- |
|  | T1 (N=49) | T2 (N=49) | SMD | T1 (N=289) | T2 (N=289) | SMD |
| Age, years |  |  | -0.1412 |  |  | 0.0612 |
| Continue (median, IQR) | 70.00 (63.00-77.00) | 69.00 (62.00-77.00) |  | 72.00 (64.00-79.00) | 72.00 (66.50-79.00) |  |
| Sex |  |  | 0.0000 |  |  | -0.0237 |
| Male | 36 (73.5) | 36 (73.5) |  | 217 (75.1) | 220 (76.1) |  |
| Female | 13 (26.5) | 13 (26.5) |  | 72 (24.9) | 69 (23.9) |  |
| Race |  |  | 0.0966 |  |  | -0.0467 |
| Caucasian | 47 (95.9) | 45 (91.8) |  | 268 (92.7) | 275 (95.2) |  |
| African | 2 (4.1) | 4 (8.2) |  | 14 (4.8) | 6 (2.1) |  |
| Other | 0 (0.0) | 0 (0.0) |  | 7 (2.4) | 8 (2.8) |  |
| Radiotherapy |  |  | 0.1016 |  |  | -0.0084 |
| Not performed | 43 (87.8) | 41 (83.7) |  | 235 (81.3) | 236 (81.7) |  |
| Performed | 6 (12.2) | 8 (16.3) |  | 54 (18.7) | 53 (18.3) |  |
| Chemotherapy |  |  | -0.1228 |  |  | 0.0209 |
| Not performed | 21 (42.9) | 24 (49.0) |  | 126 (43.6) | 123 (42.6) |  |
| Performed | 28 (57.1) | 25 (51.0) |  | 163 (56.4) | 166 (57.4) |  |
| Histology |  |  | -0.0406 |  |  | -0.1197 |
| Sarcomatoid | 1 (2.0) | 8 (16.3) |  | 27 (9.3) | 34 (11.8) |  |
| Epithelioid | 33 (67.3) | 22 (44.9) |  | 144 (49.8) | 147 (50.9) |  |
| Biphasic | 2 (4.1) | 5 (10.2) |  | 32 (11.1) | 39 (13.5) |  |
| Mesothelioma NOS | 13 (26.5) | 14 (28.6) |  | 86 (29.8) | 69 (23.9) |  |
| Grade |  |  | 0.1234 |  |  | -0.0861 |
| Well | 1 (2.0) | 0 (0.0) |  | 2 (0.7) | 5 (1.7) |  |
| Moderate | 1 (2.0) | 1 (2.0) |  | 4 (1.4) | 1 (0.3) |  |
| Poor | 1 (2.0) | 1 (2.0) |  | 9 (3.1) | 15 (5.2) |  |
| Undifferentiated | 1 (2.0) | 0 (0.0) |  | 3 (1.0) | 6 (2.1) |  |
| Unknown | 45 (97.2) | 47 (95.9) |  | 271 (93.8) | 262 (90.7) |  |
| N category |  |  | 0.0872 |  |  | 0.000 |
| 0 |  |  |  | 245 (84.8) | 245 (84.8) |  |
| 1 | 42 (85.7) | 40 (81.6) |  | 42 (14.5) | 42 (14.5) |  |
| 2 | 7 (14.3) | 9 (18.4) |  | 2 (0.7) | 2 (0.7) |  |
| Pleural effusion |  |  | 0.1309 |  |  | 0.0377 |
| Without | 6 (12.2) | 3 (6.1) |  | 87 (30.1) | 82 (28.4) |  |
| With | 43 (87.8) | 46 (93.9) |  | 202 (69.9) | 207 (71.6) |  |

SMD, standard mean difference; PSM, propensity score matching; IQR, interquartile range; NOS, not otherwise specific

**Table S2. Univariable Cox analyses in T1-2 (9^th^) patients after matching**

| Characteristic | OS | | | CSS | | |
| --- | --- | --- | --- | --- | --- | --- |
|  | HR | 95% CI | P | HR | 95% CI | P |
| Age |  |  | 0.014 |  |  | 0.021 |
| Continue | 1.030 | 1.006-1.055 |  | 1.029 | 1.004-1.055 |  |
| Sex |  |  | 0.077 |  |  | 0.116 |
| Male | 1 |  |  | 1 |  |  |
| Female | 0.630 | 0.377-1.051 |  | 0.654 | 0.385-1.110 |  |
| Race |  |  | 0.464 |  |  | 0.341 |
| Caucasian | 1 |  |  | 1 |  |  |
| African | 1.367 | 0.692-3.154 |  | 1.503 | 0.649-3.480 |  |
| Marital status |  |  | 0.183 |  |  | 0.245 |
| Other | 1 |  |  | 1 |  |  |
| Married | 0.736 | 0.468-1.156 |  | 0.684 | 0.429-1.091 |  |
| Income, dollar |  |  | 0.739 |  |  | 0.451 |
| <= 80,000 | 1 |  |  | 1 |  |  |
| > 80,000 | 1.076 | 0.699-1.657 |  | 1.188 | 0.759-1.859 |  |
| Residence |  |  | 0.558 |  |  | 0.384 |
| Urban | 1 |  |  | 1 |  |  |
| Rural | 0.740 | 0.270-2.028 |  | 0.598 | 0.188-1.902 |  |
| Radiotherapy |  |  | 0.545 |  |  | 0.359 |
| Not performed | 1 |  |  | 1 |  |  |
| Performed | 1.202 | 0.662-2.182 |  | 1.325 | 0.726-2.416 |  |
| Chemotherapy |  |  | 0.796 |  |  | 0.928 |
| Not performed | 1 |  |  | 1 |  |  |
| Performed | 0.945 | 0.614-1.454 |  | 0.979 | 0.624-1.537 |  |
| Histology |  |  | < 0.001 |  |  | < 0.001 |
| Sarcomatoid | 1 |  |  | 1 |  |  |
| Epithelioid | 0.333 | 0.153-0.726 |  | 0.309 | 0.141-0.678 |  |
| Biphasic | 1.515 | 0.547-4.194 |  | 1.534 | 0.554-4.248 |  |
| Mesothelioma NOS | 0.588 | 0.262-1.318 |  | 0.528 | 0.232-1.202 |  |
| Grade |  |  | 0.246 |  |  | 0.332 |
| Well | 1 |  |  | 1 |  |  |
| Moderate | 0.339 | 0.030-3.869 |  | 0.185 | 0.011-3.037 |  |
| Poor | 0.375 | 0.033-4.323 |  | 0.402 | 0.035-4.629 |  |
| Undifferentiated | 0.134 | 0.008-2.251 |  | 0.147 | 0.009-2.462 |  |
| Unknown | 0.159 | 0.021-1.207 |  | 0.162 | 0.021-1.230 |  |
| Laterality |  |  | 0.265 |  |  | 0.246 |
| Right | 1 |  |  | 1 |  |  |
| Left | 0.779 | 0.502-1.209 |  | 0.761 | 0.481-1.206 |  |
| T category (9^th^ edition) |  |  | 0.001 |  |  | 0.001 |
| 1 | 1 |  |  | 1 |  |  |
| 2 | 2.092 | 1.350-3.243 |  | 2.205 | 1.393-3.491 |  |
| N category |  |  | 0.673 |  |  | 0.632 |
| 0 | 1 |  |  | 1 |  |  |
| 1 | 1.125 | 0.651-1.944 |  | 1.148 | 0.652-2.023 |  |
| Pleural effusion |  |  | 0.001 |  |  | 0.002 |
| Without | 1 |  |  | 1 |  |  |
| With | 5.530 | 1.967-15.547 |  | 6.780 | 2.075-22.155 |  |

NOS, not otherwise specific; HR, hazard ratio; CI, confidence interval

**Table S3. Univariable Cox analyses in T1-2 (8^th^) patients after matching**

| Characteristic | OS | | | CSS | | |
| --- | --- | --- | --- | --- | --- | --- |
|  | HR | 95% CI | P | HR | 95% CI | P |
| Age |  |  | < 0.001 |  |  | < 0.001 |
| Continue | 1.037 | 1.027-1.047 |  | 1.034 | 1.023-1.044 |  |
| Sex |  |  | 0.004 |  |  | 0.012 |
| Male | 1 |  |  | 1 |  |  |
| Female | 0.734 | 0.593-0.908 |  | 0.753 | 0.603-0.940 |  |
| Race |  |  | 0.283 |  |  | 0.373 |
| Caucasian | 1 |  |  | 1 |  |  |
| African | 1.308 | 0.816-2.098 |  | 1.268 | 0.769-2.093 |  |
| Other | 0.702 | 0.375-1.315 |  | 0.706 | 0.364-1.367 |  |
| Marital status |  |  | 0.032 |  |  | 0.048 |
| Other | 1 |  |  | 1 |  |  |
| Married | 0.815 | 0.676-0.982 |  | 0.821 | 0.674-0.999 |  |
| Income, dollar |  |  | 0.828 |  |  | 0.755 |
| <= 80,000 | 1 |  |  | 1 |  |  |
| > 80,000 | 0.980 | 0.819-1.174 |  | 1.030 | 0.853-1.244 |  |
| Residence |  |  | 0.388 |  |  | 0.469 |
| Urban | 1 |  |  | 1 |  |  |
| Rural | 1.127 | 0.858-1.481 |  | 1.112 | 0.834-1.482 |  |
| Radiotherapy |  |  | < 0.001 |  |  | 0.004 |
| Not performed | 1 |  |  | 1 |  |  |
| Performed | 0.647 | 0.511-0.819 |  | 0.699 | 0.549-0.890 |  |
| Chemotherapy |  |  | 0.096 |  |  | 0.220 |
| Not performed | 1 |  |  | 1 |  |  |
| Performed | 0.859 | 0.719-1.027 |  | 0.889 | 0.737-1.073 |  |
| Histology |  |  | < 0.001 |  |  | < 0.001 |
| Sarcomatoid | 1 |  |  | 1 |  |  |
| Epithelioid | 0.402 | 0.298-0.544 |  | 0.399 | 0.291-0.548 |  |
| Biphasic | 0.783 | 0.546-1.124 |  | 0.822 | 0.564-1.196 |  |
| Mesothelioma NOS | 0.594 | 0.432-0.815 |  | 0.587 | 0.420-0.819 |  |
| Grade |  |  | 0.032 |  |  | 0.082 |
| Well | 1 |  |  | 1 |  |  |
| Moderate | 6.854 | 1.828-25.702 |  | 6.228 | 1.385-28.010 |  |
| Poor | 3.386 | 1.280-8.955 |  | 3.815 | 1.302-11.179 |  |
| Undifferentiated | 3.227 | 1.053-9.887 |  | 3.490 | 1.019-11.952 |  |
| Unknown | 2.501 | 1.034-6.051 |  | 2.798 | 1.043-7.507 |  |
| Laterality |  |  | 0.107 |  |  | 0.189 |
| Right | 1 |  |  | 1 |  |  |
| Left | 0.824 | 0.687-0.988 |  | 0.837 | 0.692-1.013 |  |
| Other | 0.805 | 0.332-1.949 |  | 0.893 | 0.368-2.165 |  |
| T category (8^th^ edition) |  |  | 0.057 |  |  | 0.053 |
| 1 | 1 |  |  | 1 |  |  |
| 2 | 1.186 | 0.995-1.415 |  | 1.200 | 0.997-1.443 |  |
| N category |  |  | 0.746 |  |  | 0.765 |
| 0 | 1 |  |  | 1 |  |  |
| 1 | 1.005 | 0.786-1.286 |  | 1.090 | 0.848-1.401 |  |
| 2 | 1.469 | 0.548-3.939 |  | 1.204 | 0.386-3.755 |  |
| Pleural effusion |  |  | < 0.001 |  |  | < 0.001 |
| Without | 1 |  |  | 1 |  |  |
| With | 1.914 | 1.540-2.379 |  | 2.047 | 1.621-2.585 |  |

NOS, not otherwise specific; HR. hazard ratio; CI, confidence interval
